# Supplementary material for: The Evolution and Characterization of the RNA Interference Pathways in Lophotrochozoa
Source: Genome Biol Evol. 2024 May 7;16(5):evae098. doi: 10.1093/gbe/evae098 (PMC11114477; doi:10.1093/gbe/evae098)
Supplement: evae098_Supplementary_Data [file evae098_supplementary_data.zip › Supplementary_materials.zip!/selected_miRNAs/DaRe_selected_miRNas/DaRe_NW_018394463.1_42257.pdf]

Provisional ID	:	NW_018394463.1_42257
Score total	:	258.1
Score for star read(s)	:	3.9
Score for read counts	:	250.5
Score for mfe	:	2.8
Score for randfold	:	1.6
Score for cons. seed	:	-0.6
Total read count	:	503
Mature read count	:	496
Loop read count	:	0
Star read count	:	7

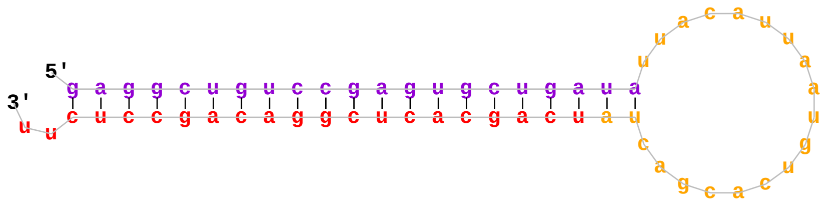

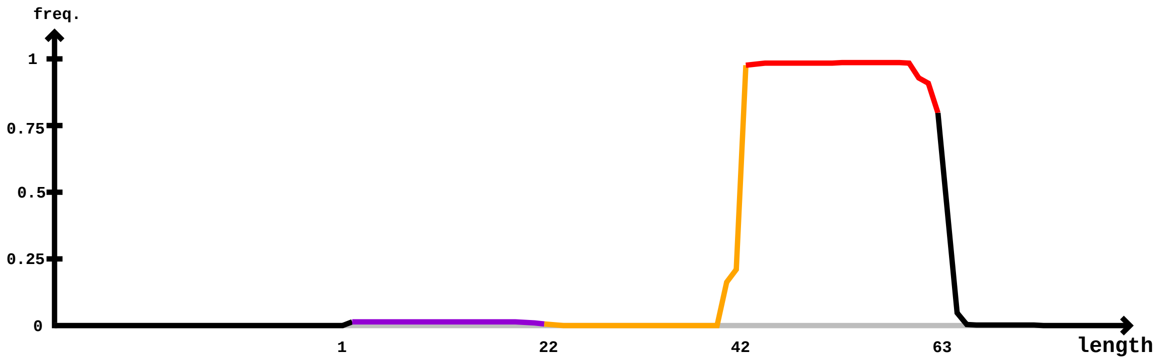


```
                                  Star                                      Mature
5'- guucucgaaucugauugcacaagggugaagaggcuguccgagugcugauauuacauuaaugucacgacuaucagcacucggacagccucuucacccuugugcauuacucc  -3'   obs
    guucucgaaucugauugcacaagggugaagaggcuguccgagugcugauauuacauuaaugucacgacuaucagcacucggacagccucuucacccuugugcauuacucc         exp
    ...............(((((((((((((((((((((((((((((((((((..................))))))))))))))))))))))))))))))))))).......   reads   mm   sample
    ....................................................................uaucagcacucggacagccu......................   2       0    Ov3
    ....................................................................uaucagcacucggacagccG......................   1       1    Ov3
    ....................................................................uaucagcacucggacagccuc.....................   2       0    Ov3
    ....................................................................uaucagcacucggacagccucA....................   2       1    Ov3
    ....................................................................uaucagcacucggacagccucu....................   8       0    Ov3
    ....................................................................uaucagcacucggacagccucC....................   1       1    Ov3
    ....................................................................uaucagcacucggacagccucuu...................   1       0    Ov3
    .....................................................................aucagcacucggacagccucuu...................   2       0    Ov3
    .....................................................................aucagcacucggacagccucuA...................   1       1    Ov3
    ......................................................................ucagcacucggacagccu......................   1       0    Ov3
    ......................................................................ucagcacucggacagccucu....................   4       0    Ov3
    ......................................................................ucagcacucggacagccucuC...................   1       1    Ov3
    ......................................................................ucagcacucggacagccucuu...................   51      0    Ov3
    ......................................................................ucagcacucggacagccucuuU..................   5       1    Ov3
    ......................................................................ucagcacucggacagccucuuc..................   1       0    Ov3
    ......................................................................ucagcacucggacagccucuuA..................   13      1    Ov3
    ......................................................................ucagcacucggacagccucuuAa.................   2       1    Ov3
    ......................................................................ucagcacucggacagccucuCca.................   3       1    Ov3
    ......................................................................ucagcacucggacagccucuuca.................   1       0    Ov3

    .............................gaggcuguccgagugcug...............................................................   1       0    Gu1
    .............................gaggcuguccgagugcugauU............................................................   2       1    Gu1
    .............................gaggcuguUcgagugcugaua............................................................   1       1    Gu1
    ....................................................................uaucagcacucggacagcc.......................   1       0    Gu1
    ....................................................................uaucagcacucggacagccu......................   10      0    Gu1
    ....................................................................uaucagcacucggacagccuc.....................   2       0    Gu1
    ....................................................................uaucagcacucggacagccucu....................   12      0    Gu1
    ....................................................................uaucagcacucggacagccucC....................   1       1    Gu1
    ....................................................................uaucagcacucggacagccucuu...................   1       0    Gu1
    .....................................................................aucagcacucggacagccu......................   1       0    Gu1
    .....................................................................aucagcacucggacagccuc.....................   1       0    Gu1
    .....................................................................aucagcacucggacagccucu....................   1       0    Gu1
    .....................................................................aucagcacucggacagccucuA...................   1       1    Gu1
```

```
                             Star                                     Mature
guucucgaaucugauugcacaagggugaagaggcuguccgagugcugauauuacauuaaugucacgacuaucagcacucggacagccucuucacccuugugcauuacucc
.....................................................................aucagcacucggacagccucuC...................   1   1   Gu1
.....................................................................aucagcacucggacagccucuu...................   3   0   Gu1
.....................................................................aucagcacucggacagccucuuA..................   1   1   Gu1
.....................................................................aucagcacucggacagccucuuU..................   1   1   Gu1
......................................................................ucagcacucggacagccu......................   1   0   Gu1
......................................................................ucagcacucggacagccuc.....................   1   0   Gu1
......................................................................ucagcacucggacagccucu....................   3   0   Gu1
......................................................................ucagcacucggGcagccucuu...................   1   1   Gu1
......................................................................ucagcacucggacagccucuA...................   1   1   Gu1
......................................................................ucagcacucggacagccucuu...................   48  0   Gu1
......................................................................ucagcacucggacagccucuuU..................   16  1   Gu1
......................................................................ucagcacucggacagccucuuA..................   19  1   Gu1
......................................................................ucagcacucggacagccucuuc..................   1   0   Gu1
......................................................................ucagcacucggacagccucuuUa.................   1   1   Gu1
......................................................................ucagcacucggacagccucuuAa.................   2   1   Gu1
.......................................................................cagcacucggacagccucuucC.................   2   1   Gu1

.............................gaggcuguUcgagugcugau.............................................................   1   1   Gu2
....................................................................uaucagcacucAgacagccu......................   1   1   Gu2
....................................................................uaucagcacucggacagccu......................   4   0   Gu2
....................................................................uaucagcacucggacagccuc.....................   2   0   Gu2
....................................................................uaucagcacucggacagccucu....................   6   0   Gu2
....................................................................uaucagcacucggacagccucuA...................   1   1   Gu2
....................................................................uaucagcacucggacagccucuu...................   1   0   Gu2
.....................................................................aucagcacucggacagccucuu...................   1   0   Gu2
.....................................................................aucagcacucggacagccucuC...................   1   1   Gu2
.....................................................................aucagcacucggacagccucuuU..................   1   1   Gu2
.....................................................................aucagcacucggacagccucuuAac................   1   1   Gu2
......................................................................ucagcacucggacagccu......................   2   0   Gu2
......................................................................ucagcacucggacagccucu....................   1   0   Gu2
......................................................................ucagcacucggacagccucuu...................   46  0   Gu2
......................................................................ucagcacucggacagccucuC...................   1   1   Gu2
......................................................................ucagcacucggacagccucuuA..................   5   1   Gu2
......................................................................ucagcacucggacagccucuuU..................   5   1   Gu2
......................................................................ucagcacucggacagccucuuAa.................   2   1   Gu2

....................................................................uaucagcacucggacagccu......................   1   0   Ov2
....................................................................uaucagcacucggacagccuc.....................   1   0   Ov2
....................................................................uaucagcacucggacagccucu....................   4   0   Ov2
.....................................................................auAagcacucggacagccucuu...................   1   1   Ov2
.....................................................................aucagcacucggacagccucuu...................   1   0   Ov2
.....................................................................aucagcacucggacagccucuuU..................   2   1   Ov2
......................................................................ucagcacucggacagccucu....................   1   0   Ov2
......................................................................ucagcacucgAacagccucuu...................   1   1   Ov2
......................................................................ucagcacucggacagccucuu...................   34  0   Ov2
......................................................................ucagcacucggacagccucuuc..................   2   0   Ov2
......................................................................ucagcacucggacagccucuCc..................   1   1   Ov2
......................................................................ucagcacucggacagccucuuU..................   3   1   Ov2
......................................................................ucagcacucggacagccucuuA..................   2   1   Ov2
......................................................................ucagcacucggacagccucuuAa.................   1   1   Ov2
........................................................................agcacucggacagccucuu...................   1   0   Ov2

.............................gaggcuguccgagugcuga..............................................................   1   0   Gu3
.............................gaggcuguUcgagugcugau.............................................................   1   1   Gu3
....................................................................uaucagcacucggacagccu......................   2   0   Gu3
....................................................................uaucagcacucggacagccucu....................   7   0   Gu3
....................................................................uaucagcacucggacagccucA....................   1   1   Gu3
....................................................................uaucagcacucggacagccucuu...................   2   0   Gu3
.....................................................................aucagcacucggacagccucuu...................   2   0   Gu3
......................................................................ucagcacucggacagccucu....................   2   0   Gu3
......................................................................ucagcacucggacagccucuu...................   47  0   Gu3
......................................................................ucagcacucggacagccuAuu...................   1   1   Gu3
......................................................................ucagcacucggacagccucuuU..................   10  1   Gu3
......................................................................ucagcacucggacagccucuuA..................   4   1   Gu3
......................................................................ucagcacucggacagccucuuUa.................   1   1   Gu3
......................................................................ucagcacucggacagccucuuAa.................   1   1   Gu3
........................................................................agcacucggacagccucuu...................   1   0   Gu3

....................................................................uaucagcacucgAacagccu......................   1   1   Ov1
....................................................................uaucagcacucggacagccuc.....................   1   0   Ov1
```

```
                            Star                                        Mature
guucucgaaucugauugcacaagggugaagaggcuguccgagugcugauauuacauuaaugucacgacuaucagcacucggacagccucuucacccuugugcauuacucc
....................................................................uaucagcacucggacagccucu....................    2     0     Ov1
....................................................................uaucagcacucggacagccucuA...................    1     1     Ov1
.....................................................................aucagcacucggacagccucuuU..................    1     1     Ov1
......................................................................ucagcacucggacagccu......................    1     0     Ov1
......................................................................ucagcacucggacagccucuu...................    18    0     Ov1
......................................................................ucagcacucggacagccucuuA..................    5     1     Ov1
......................................................................ucagcacucggacagccucuCc..................    1     1     Ov1
......................................................................ucagcacucggacagccucuuc..................    3     0     Ov1
......................................................................ucagcacucggacagccucuuU..................    3     1     Ov1
......................................................................ucagcacucggacagccucuCca.................    2     1     Ov1
......................................................................ucagcacucggacagccucuuAa.................    4     1     Ov1
................................................................................Uacagccucuucacccuugug.........    1     1     Ov1
```
